# Polymorphous Cutaneous Sarcoidosis With Excellent Response to Minocycline

**DOI:** 10.7759/cureus.47902

**Published:** 2023-10-29

**Authors:** Pranvera Sulejmani, Emily J Medhus, Pamela N Madu, Kyle T Amber

**Affiliations:** 1 Dermatology, Rush University Medical Center, Chicago, USA

**Keywords:** polymorphous, minocycline, darier-roussy, lupus pernio, sarcoidosis

## Abstract

Sarcoidosis is notorious for producing a wide variety of skin lesions, which are categorized as either specific or nonspecific. The specific lesions include primary morphologies ranging from micropapules to subcutaneous nodules. Nonspecific skin lesions include associated conditions like erythema nodosum, calcinosis cutis, and prurigo. It is not uncommon for a patient to have a combination of specific and nonspecific lesions. In contrast, it is exceedingly rare for one patient to have multiple specific sarcoidal lesions. When present, the term “polymorphous cutaneous sarcoidosis” has been used. We present the case of a patient who presented with three specific cutaneous morphologies of sarcoidosis: papular sarcoid, Darier-Roussy subcutaneous sarcoidosis, and lupus pernio. After only two months of oral minocycline, our patient demonstrated remarkable improvement with near-complete resolution of the cutaneous lesions. In addition to describing the rare polymorphous presentation, this case also highlights the challenge of relating lesion type to overall prognosis when multiple morphologies are present.

## Introduction

There is a wide range of cutaneous manifestations of sarcoidosis. Many different morphologies have been described, including papules, plaques, subcutaneous nodules, lupus pernio, annular lesions, scar-associated, psoriasiform, and so on. While many patients with sarcoidosis will have some form of skin involvement, it is exceedingly rare to develop more than one morphology of cutaneous sarcoidosis in a single patient [1]. To our knowledge, there are only a handful of cases previously reported in the literature [2-4]. A primary sarcoidal morphology in combination with nonspecific manifestations of sarcoid, such as erythema nodosum, are, in contrast, well described [5,6]. In cases where multiple primary manifestations of sarcoid are present, the term “polymorphous sarcoid” has been used in the literature [2]. We present the case of a patient who presented with three different specific cutaneous morphologies of sarcoidosis, without a prior history or diagnosis of sarcoidosis, and highlight her remarkable response to oral minocycline.

## Case presentation

A 65-year-old female with a past medical history of hypertension presented for evaluation of lesions on her arms and around her eyes. Asymptomatic subcutaneous nodules on her volar forearms appeared six months prior to presentation with a gradual increase in lesions over time. Moreover, during that time, she developed papules around her eyes, which were occasionally painful and caused significant eye irritation. On further review, she mentioned a rash on her neck that had been present for a longer time, unclear of exactly how long, but did not think it was of any significance. She denied arthralgias, cough, or shortness of breath. Family history was non-contributory.

**Figure 1:**
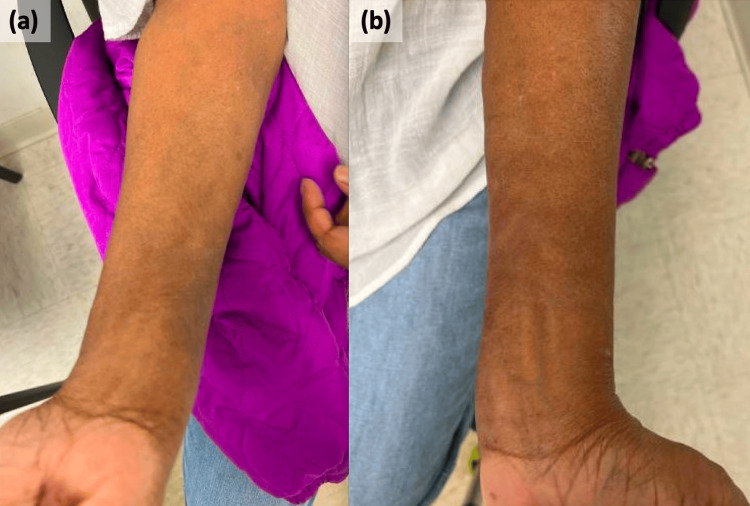
Many subcutaneous nodules involving the right (a) and left (b) volar forearms without overlying erythema or skin surface changes.

The examination was notable for numerous non-tender, discrete, firm subcutaneous nodules without overlying erythema or epidermal change on her bilateral volar forearms (Figure 1). Sensation to light touch was intact on the upper extremities, and she denied numbness or tingling.

**Figure 2:**
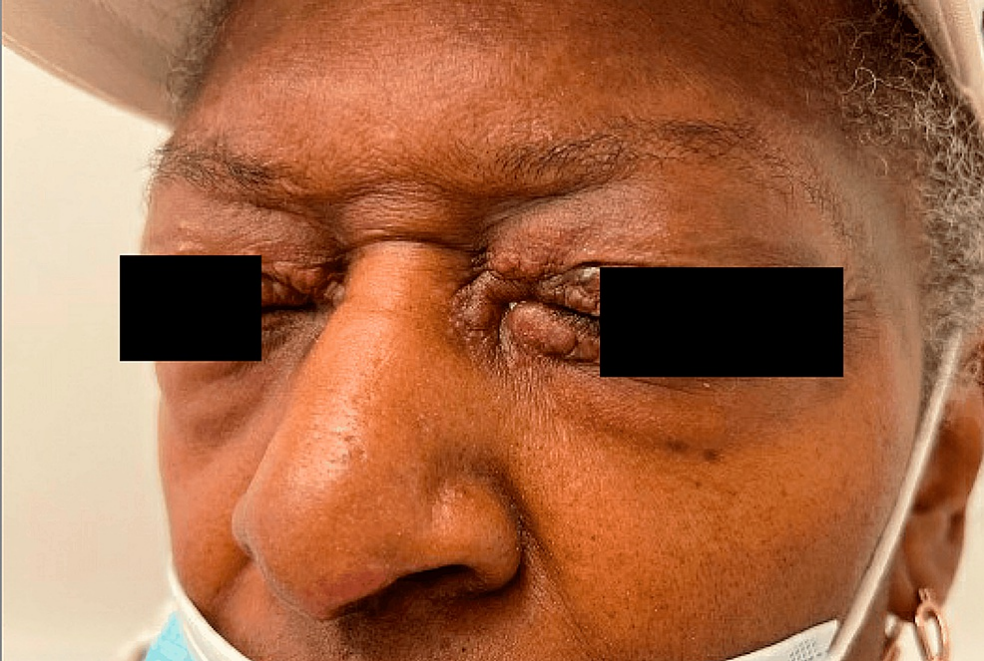
Erythematous papules on the medial canthus (left > right) and lupus pernio on the left nasal alar rim.

Small, slightly erythematous papules, some coalescing to plaques, were present on the medial canthi and eyelids, and a small papule was appreciated on the nasal tip (Figure 2).

**Figure 3:**
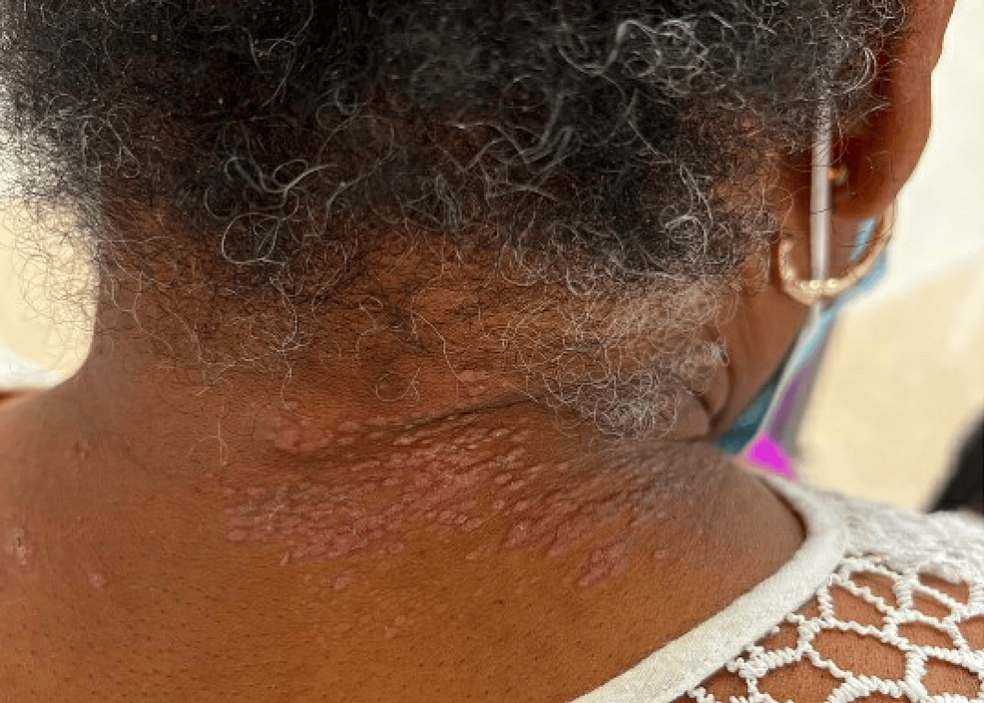
Erythematous papules coalescing into plaques on the posterior right neck.

Lastly, on the posterior right neck, there were many 1-2 mm erythematous papules coalescing into plaques (Figure 3). The patient’s torso and lower extremities were unaffected.

**Figure 4:**
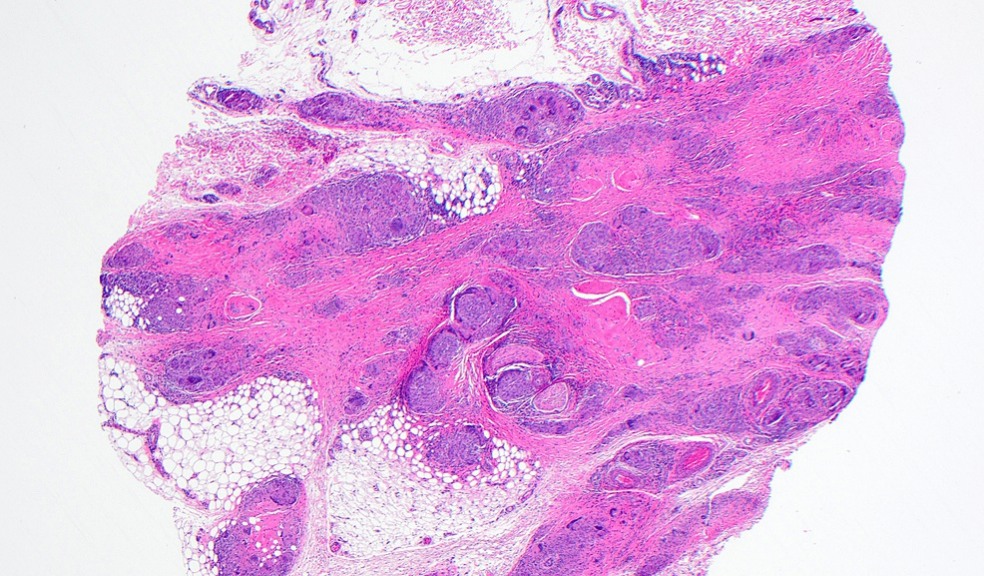
Histology findings (H&E) from the left anterior forearm at 20x magnification showing nodular aggregates of multinucleated epithelioid histiocytes with a thin rim of lymphocytes in the subcutaneous tissue.

**Figure 5:**
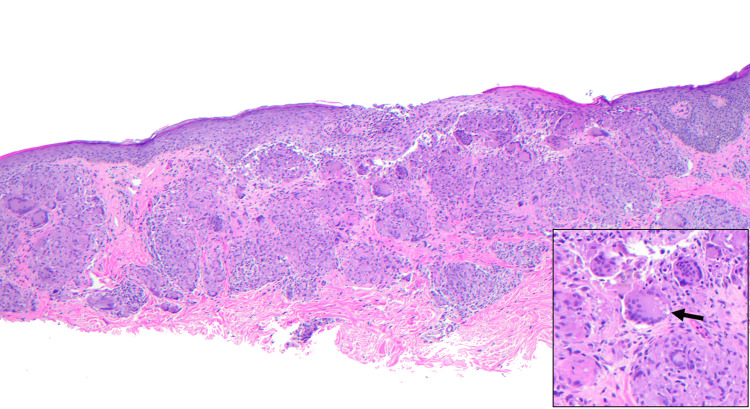
H&E findings from the posterior neck at 40x magnification showing granulomatous dermatitis within the dermis. The inset shows an asteroid body (arrow) at 100x magnification.

Two biopsies were obtained: a shave biopsy from the posterior neck and a telescoping punch biopsy from the left anterior forearm. Given the clinical suspicion of sarcoidosis from the other sites, eyelid and nose biopsies were deferred. Both biopsies showed nodular aggregates of granulomatous inflammation (Figures 4-5), no evidence of a foreign body with a polarizing lens, and negative AFB and PAS-D immunohistochemistry. An asteroid body was present in the biopsy specimen from the posterior neck (Figure 5.) The histologic findings and special stain results supported a diagnosis of sarcoidosis.

**Figure 6:**
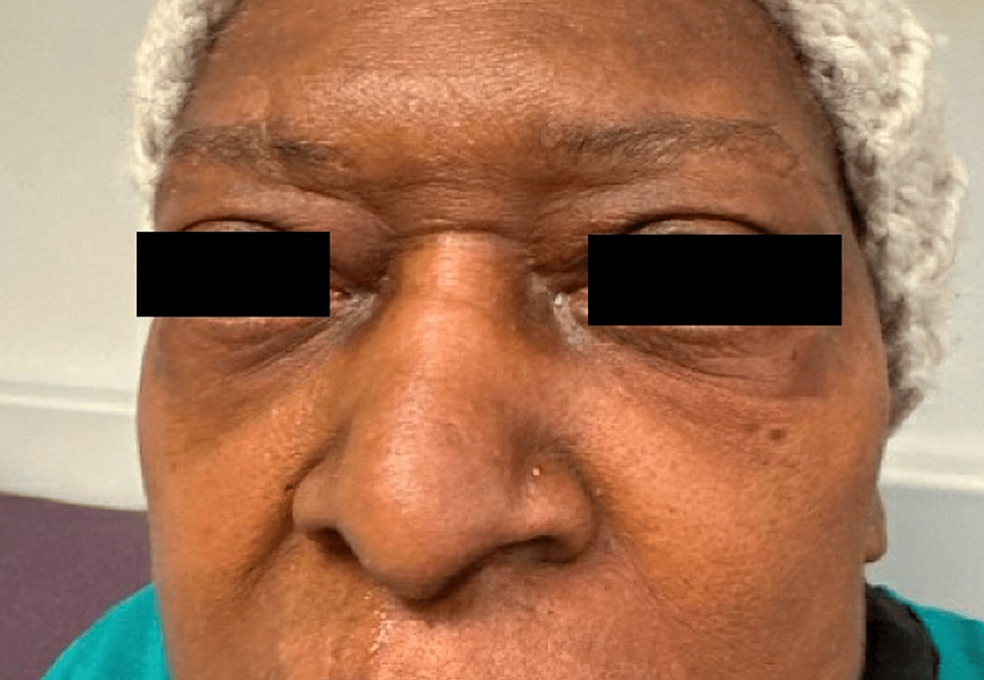
Near complete resolution of all lesions on the face after two months of oral minocycline.

**Figure 7:**
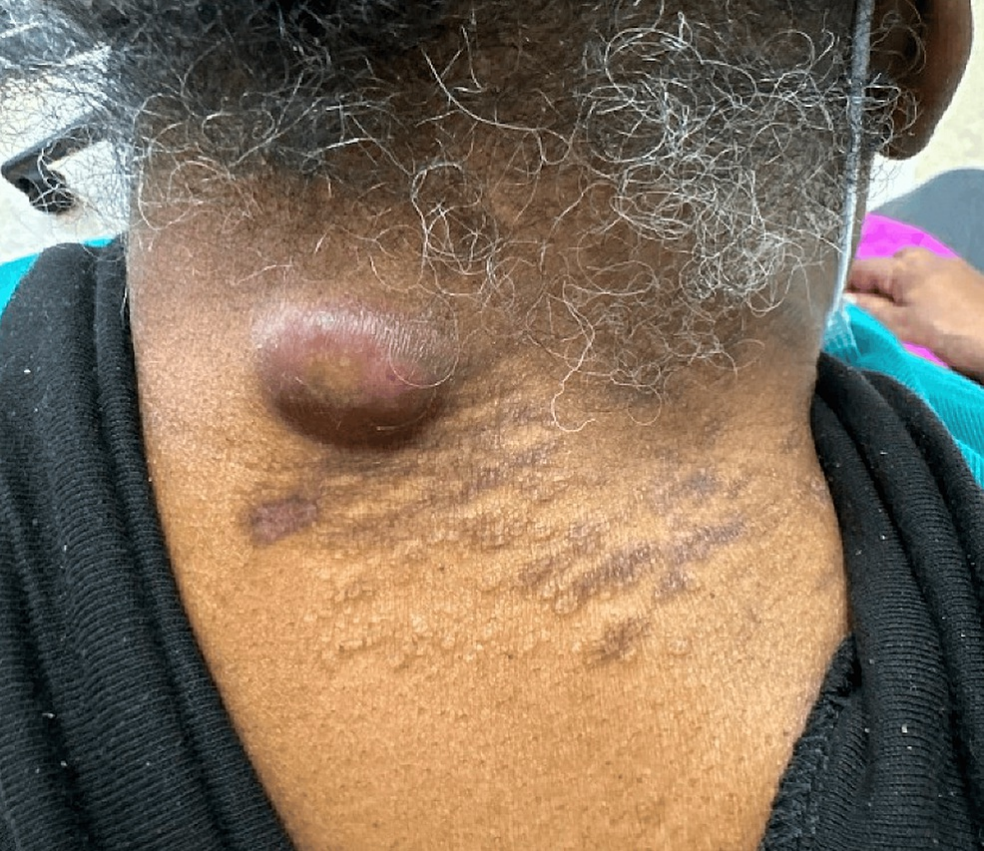
Post-treatment clinical findings on the posterior neck, notable for post-inflammatory hyperpigmentation and an unrelated inflamed cyst.

The patient was started on oral minocycline 100 mg twice a day and referred to rheumatology for further systemic workup. Labs were notable for an elevated angiotensin-converting enzyme of 233 U/L. A chest X-ray and CT were recommended but not completed by the patient. She was also noted to have elevated creatinine but declined further investigations. After two months on oral minocycline, she returned for follow-up and showed significant improvement with near-complete resolution of all lesions (Figures 6-7). 

## Discussion

While the lungs are the most commonly affected organ in sarcoidosis, it is not uncommon for cutaneous sarcoidosis to be the presenting sign of sarcoidosis [6,7]. When present, cutaneous findings can range in appearance and morphology; papules/plaques and subcutaneous nodules being the most common, with lupus pernio considered less common [5,8]. Specific skin lesions are classified as such based on the presence of non-caseating granulomas on histology as opposed to nonspecific lesions, which do not have granuloma formation [2,3,6]. Associations have been made between skin lesion morphology and risk of systemic involvement; therefore, lesion type can be helpful in predicting prognosis [6]. It is not uncommon for a patient to have a combination of specific and nonspecific skin lesions, but it is rare for multiple specific lesions to occur simultaneously in a single patient [1].

There are only a few reports of polymorphous cutaneous sarcoidosis in the literature to date. Interestingly, the reported cases all present different combinations of lesion morphologies, and underlying systemic involvement seems arbitrary. The first case described a combination of papular, psoriasiform, and pigmented purpuric dermatosis-like lesions in a 31-year-old male from Bangladesh [3]. This patient did not appear to have signs or symptoms of systemic involvement. Another case involved the presence of subcutaneous nodules, annular plaques, and erythema nodosum-like lesions in a 56-year-old woman [2]. This patient had known pulmonary sarcoidosis and bilateral hilar lymphadenopathy before the development of these cutaneous lesions. Finally, the combination of eyelid papules, palmar nodules, and scar/tattoo sarcoid was described in a 30-year-old woman with widespread systemic involvement affecting multiple organ systems [4].

Historically, the type of skin lesion has been used as a marker for prognosis; however, polymorphous cutaneous sarcoidosis poses a challenge to that concept [6]. When multiple lesions are present, can the same rules be applied? For example, one of the lesions present in our patient was subcutaneous nodules, which would indicate a good prognosis and is usually associated with non-severe systemic involvement [6]. However, she also had lupus pernio lesions, which are frequently associated with sarcoidosis of the lungs and upper respiratory tract [6]. The ability to use lesion morphology to predict possible systemic involvement or disease course is compromised when multiple morphologies are present.

The ultimate outcome for our patient is difficult to determine as the patient declined the recommended systemic workup. Systemic involvement seems unlikely based on the lack of symptoms, but pulmonary, hilar lymph node, and renal involvement cannot be completely ruled out at this time.

## Conclusions

Polymorphous cutaneous sarcoidosis describes the presence of multiple specific sarcoidal lesions occurring in one patient. This phenomenon is rarely reported in the literature. Our case of polymorphous cutaneous sarcoidosis demonstrates the unique combination of sarcoid specific lesions not previously reported: papular sarcoid, Darier-Roussy subcutaneous sarcoidosis, and lupus pernio. Fortunately, our patient demonstrated remarkable improvement with a near-complete resolution of the cutaneous lesions, after only two months of oral minocycline.
